# Apolipoprotein A-IV is induced by high-fat diets and mediates positive effects on glucose and lipid metabolism

**DOI:** 10.1016/j.molmet.2025.102119

**Published:** 2025-03-01

**Authors:** Anne-Marie Lundsgaard, Rita Del Giudice, Josephine M. Kanta, Mark Larance, Sarah L. Armour, Amalie London, Michael M. Richter, Nicoline R. Andersen, Trine S. Nicolaisen, Christian S. Carl, Kim A. Sjøberg, Kirstine Nyvold Bojsen-Møller, Jakob G. Knudsen, Jens O. Lagerstedt, Andreas M. Fritzen, Bente Kiens

**Affiliations:** 1 The August Krogh Section for Human & Molecular Physiology, Department of Nutrition, Exercise and Sports, Faculty of Science, University of Copenhagen, Copenhagen, Denmark; 2 Novo Nordisk A/S, Søborg, Denmark; 3 Department of Experimental Medical Science, Lund University, Lund, Sweden; 4 Department of Biomedical Science and Biofilms - Research Center for Biointerfaces, Malmö University, Malmö, Sweden; 5 Charles Perkins Centre and School of Medical Sciences, University of Sydney, Sydney, Australia; 6 Section for Cell Biology and Physiology, Department of Biology, Faculty of Science, University of Copenhagen, Copenhagen, Denmark; 7 Department of Endocrinology, Copenhagen University Hospital Hvidovre, Hvidovre, Denmark; 8 Department of Clinical Biochemistry, Copenhagen University Hospital Bispebjerg and Frederiksberg, Copenhagen, Denmark; 9 Department of Biomedical Sciences, Faculty of Health and Medical Sciences, University of Copenhagen, Copenhagen, Denmark; 10 Department of Clinical Medicine, University of Copenhagen, Copenhagen, Denmark; 11 Department of Clinical Sciences, Lund University Diabetes Centre, Malmö, Sweden

**Keywords:** Chylomicron, Incretin hormone, Insulin secretion, Fatty acid oxidation, Hepatic glucose production, Adipose tissue, Liver, Diet

## Abstract

**Objective:**

Low-carbohydrate, high-fat diets under eucaloric conditions are associated with several health-beneficial metabolic effects in humans, particularly in the liver. We recently observed that apolipoprotein A-IV (apoA-IV), a highly abundant apolipoprotein, was among the most upregulated proteins in circulation after six weeks of consuming a high-fat diet in humans. However, the impact of dietary changes in regulating apoA-IV, and the potential effects of apoA-IV on regulation of glucose- and lipid metabolism remain to be fully established.

**Methods:**

We investigated the regulation of circulating fasting concentrations of apoA-IV in humans in response to diets enriched in either fat or carbohydrates. Moreover, to study the whole-body and tissue-specific glucose and lipid metabolic effects of apoA-IV, we administrered apoA-IV recombinant protein to mice and isolated pancreatic islets.

**Results:**

We demonstrate that in healthy human individuals high-fat intake increased fasting plasma apoA-IV concentrations by up to 54%, while high-carbohydrate intake suppressed plasma apoA-IV concentrations. In mice, administration of apoA-IV acutely lowered blood glucose levels both in lean and obese mice. Interestingly, this was related to a dual mechanism, involving both inhibition of hepatic glucose production and increased glucose uptake into white and brown adipose tissues. In addition to an effect on hepatic glucose production, the apoA-IV-induced liver proteome revealed increased capacity for lipoprotein clearance. The effects of apoA-IV in the liver and adipose tissues were concomitant with increased whole-body fatty acid oxidation. Upon glucose stimulation, an improvement in glucose tolerance by apoA-IV administration was related to potentiation of glucose-induced insulin secretion, while apoA-IV inhibited glucagon secretion *ex vivo* in islets.

**Conclusions:**

We find that apoA-IV is potently increased by intake of fat in humans, and that several beneficial metabolic effects, previously associated with high fat intake in humans, are mimicked by administration of apoA-IV protein to mice.

## Highlights

• In humans, short- and long-term high-fat intake increase circulating apoA-IV levels.
• ApoA-IV administration lowers blood glucose levels both in lean and obese mice.
• This is via inhibition of hepatic glucose production and increased uptake in WAT & BAT.
• ApoA-IV increases fatty acid oxidation and increases lipoprotein clearance in liver.
• ApoA-IV improves glucose tolerance likely via stimulating insulin and inhibiting glucagon.

## 1 Introduction

In individuals with type 2 diabetes and/or metabolical dysfunction-associated steatotic liver disease (MASLD), low-carbohydrate, high-fat diets have been shown to lower liver triacylglycerol (TG) content, while also improving glycemic control and cardiometabolic risk factors [[1], [2], [3], [4]]. Metabolic improvements with high-fat intake in most of these studies were, however, also accompanied with weight loss, potentially contributing to the observed effects. Recently, we demonstrated that even *eucaloric* low-carbohydrate, high-fat diets (∼70E% fat enriched in either unsaturated or saturated fat) consumed for 4 days or six weeks by men with overweight or obesity maintained peripheral insulin sensitivity, increased whole-body fatty acid oxidation, reduced plasma TG levels and led metabolic improvements at the liver level, including reduced intrahepatic TG content [5] and attenuated glucose production [6]. Importantly, the molecular mechanisms orchestrating the metabolic effects of such regimens with increased fat intake remain to be elucidated. Interestingly, we observed, by proteomics analysis of fasting plasma obtained from men with obesity and also from mice, that after six weeks consumption of an eucaloric high-fat diet (64E% fat) enriched in either unsaturated or saturated fatty acids (FAs), apolipoprotein A-IV (apoA-IV) was the circulating protein exhibiting the most significant increase [6]. This leaves the question whether the beneficial metabolic effects observed after high-fat intake could be mediated by apoA-IV.

ApoA-IV is a glycoprotein, and among the ten most abundant proteins in human plasma [7]. Typical of the exchangeable apolipoproteins, the structure of apoA-IV is dynamic, as is apoA-IV´s affinity for lipid [8]. ApoA-IV is present in the circulation in a lipid-free, as well as lipid-bound form. In humans, apoA-IV mRNA is expressed in the small intestine [9] and mRNA is also reported in the liver [10], while hypothalamic apoA-IV mRNA expression is demonstrated in rodents [11].

In response to acute fat intake, plasma apoA-IV concentrations have, in healthy individuals, been shown to increase by 17%–32% 4–5 h after intake of 40–100 g fat when compared with fasting levels [9,[12], [13], [14]]. This acute increase is believed to originate from intestinal apoA-IV secreted to the blood via the mesenteric lymph on the surface of chylomicrons, from which apoA-IV is displaced, leaving the majority of apoA-IV free, while another fraction is bound to high-density lipoprotein (HDL) [9,12,15]. In contrast to the acute regulation, less is known about how altered fat content in the diet more chronically regulates fasting levels of apoA-IV.

ApoA-IV was initially described to regulate reverse cholesterol transport, by activating-lecithin-cholesterol acyltransferase (LCAT) and cholesterol ester transfer protein (CETP), and thus have anti-atherosclerotic potential [16]. Among the first described functions of apoA-IV was also the induction of satiety in mice [17]. Emerging evidence also points to an important role of apoA-IV in regulation of substrate metabolism. Given the intestinal synthesis and secretion of apoA-IV upon acute fat intake, a role in postprandial metabolism seems likely. *In vitro,* apoA-IV increases the activity of lipoprotein lipase, the key enzyme responsible for hydrolysis of circulating TG, in an apoC–II–dependent manner [18].

There is also cumulative evidence pointing towards apoA-IV contributing to regulation of glucose homeostasis. The apoA-IV knockout (KO) mouse [19] shows an impaired glucose tolerance and attenuated glucose-stimulated insulin secretion [20], though the phenotype must be carefully interpreted, as apoC-III is also absent in this mouse model [21]. Moreover, administration of mouse apoA-IV protein to KKAy mice, which resemble a MAFLD phenotype, lowered blood glucose levels and suppressed gluconeogenic genes in the liver [20,22].

In humans, an inverse association has been reported between fasting plasma concentration of apoA-IV and both fasting glucose levels and the degree of oral glucose tolerance [23]. Also, the plasma apoA-IV levels were higher in healthy individuals with obesity compared with metabolically impaired individuals with obesity [24].

The metabolic actions and the regulation of apoA-IV are, however, still to be established. We speculated that circulating fasting levels of apoA-IV are upregulated in response to changes in dietary fat intake, while downregulated with high-carbohydrate intake in humans. Moreover, it was speculated that administration of apoA-IV protein to mice would mimic the health-beneficial physiological effects observed during eucaloric high-fat diet feeding in humans, such as decreased hepatic glucose production [6].

Thus, we herein investigated the regulation of circulating fasting concentrations of apoA-IV in humans in response to diets enriched in either fat or carbohydrates. Furthermore, we took a holistic approach and investigated the effect of apoA-IV administration on glucose and lipid metabolism on both a whole-body and a tissue-specific level in mice.

## 2 Materials and methods

### 2.1 Human dietary interventions

ApoA-IV was measured in plasma obtained in the overnight-fasted state from two dietary interventions, during which carbohydrate and fat intake were manipulated in healthy individuals with with obesity.

In study 1, plasma was sampled from eleven healthy men (age of 40 ± 8 (SD)) with overweight or obesity (BMI of 32 ± 4 kg/m^2^) before and after consumption of a five day eucaloric low-carbohydrate, high-fat (HFLC) diet comprised of 14E% carbohydrate, 70E% fat, 16E% protein and a five days eucaloric high-carbohydrate, low-fat (LFHC) diet with 70E% carbohydrate, 14E% fat, 16 E% protein in randomized order, both preceded by two days of a standard controlled diet (CON) [5]. In both HFLC and LFHC, the composition of dietary fat was distributed equally between saturated (SFA), monounsaturated (MUFA), and polyunsaturated (PUFA) fat. Other data from this study has been published previously [5]. The study received approval from the Ethical Committee for the Capital Region of Denmark (H-20010659) and carried out in accordance with the Declaration of Helsinki and registered at clinicaltrials.gov (NCT04581421).

In study 2, plasma was sampled from nine healthy, young (23 ± 3 years old) lean (23.7 ± 1.7 kg/m^2^) men before and after consumption of a five day eucaloric control diet (24E% fat, 62 E% carbohydrate, 14 E% protein) and three days hypercaloric (+75% energy) high-fat, low-carbohydrate diets enriched in either unsaturated fat (HF UNSAT) (78E% fat, 10 E% carbohydrate, 12 E% protein) or saturated fat (HF SAT) (83E% fat, 9E% carbohydrate, 12E% protein) or a hypercaloric (+75% energy) high-carbohydrate, low-fat diet (80 E% carbohydrate, 11 E% protein, 9 E% fat) in randomized order. Other data from this study has been published previously [[25], [26], [27]]. The study was approved by the Copenhagen Ethics Committee (KF 01 261127).

### 2.2 Animals and diets

All animal experiments were approved by the Danish Animal Experiments Inspectorate and complied with the EU convention for the protection of vertebrate animals used for scientific purposes. Female (or male as described in fig. S3) C57BL/6JRj mice aged 14–20 weeks (Janvier Labs, FR) were used for all experiments not including transgenic mice. To investigate the role of growth/differentiation factor 15 (GDF15) in apoA-IV, GDNF family receptor α-like (GFRAL) knockout (KO) and wildtype (WT) littermates were generated as previously reported [28]. Mice were housed on a 12:12 h dark–light cycle (light on at 6 a.m.) at 22±1 °C. After arrival, mice had free access to a standard rodent chow diet (Altromin #1324, Brogaarden, DK) and were group-housed in groups of 5–8 mice. Five days prior to and during both the acute and prolonged experiments, mice were single-housed in order to avoid both the stress from taken mice from a cage with several mice during the experimental day and be able to measure food intake during prolonged studies - and at the same time also avoid the immediate stress in the hours/day after being single-housed. Eight weeks before the experiments, mice had ad libitum access to either chow, high-fat, high-sucrose (HFHS) or high-fat diet (HFD), as specified. The HFHS diet comprised 58 kcal% fat (7% soybean oil, 93% coconut oil) and 25 kcal% carbohydrate (maltodextrin and sucrose 1:1) (D12331, Research Diets, US) and the HFD had 60 kcal% fat (10% soybean oil, 90% lard) and 20 kcal% carbohydrate (maltodextrin and sucrose 2:1) (D12492, Research Diets, US). Mice were fasted for 2 h prior to the experiments.

### 2.3 Recombinant proteins

#### 2.3.1 Apolipoprotein A-IV

Human apoA-IV, containing a His-tag and tobacco etch virus (TEV) protease recognition site at the N-terminus, was expressed in the bacterial *Escherichia coli* (*E. coli*) BL21 (DE3) pLysS strain. Bacterial cells were transformed with the recombinant plasmid and cultivated at 37 °C in NZYM medium supplemented with 0.2% glucose and antibiotics. Protein expression was induced for 4 h following the addition of 1 mM isopropyl-beta-thiogalactopyranoside (IPTG), and apoA-IV was purified (fig. S1) using immobilized metal affinity chromatography (IMAC) followed by TEV protease treatment and a second IMAC to remove the His-tag. Endotoxin removal was performed by using 0.5 ml high-capacity endotoxin removal columns (Pierce #82274, Thermo Fisher Scientific, US). The endotoxin quantification was performed by using Chromogenic Endotoxin Quant Kit (Pierce #A39552S, Thermo Fisher Scientific, US), revealing values below 0.8 EU/ml. The recombinant apoA-IV protein was assessed for its lipid clearance capacity and cholesterol efflux ability as described [29].

ApoA-IV was administrated by intraperitoneal injection at 1 mg/kg body weight (BW), except for the experiment in Figure 4G–I (0.5 mg/kg BW) (Figure 4), diluted in 10 μl saline per gram BW. Saline was administrated as vehicle.

**Figure 1:**
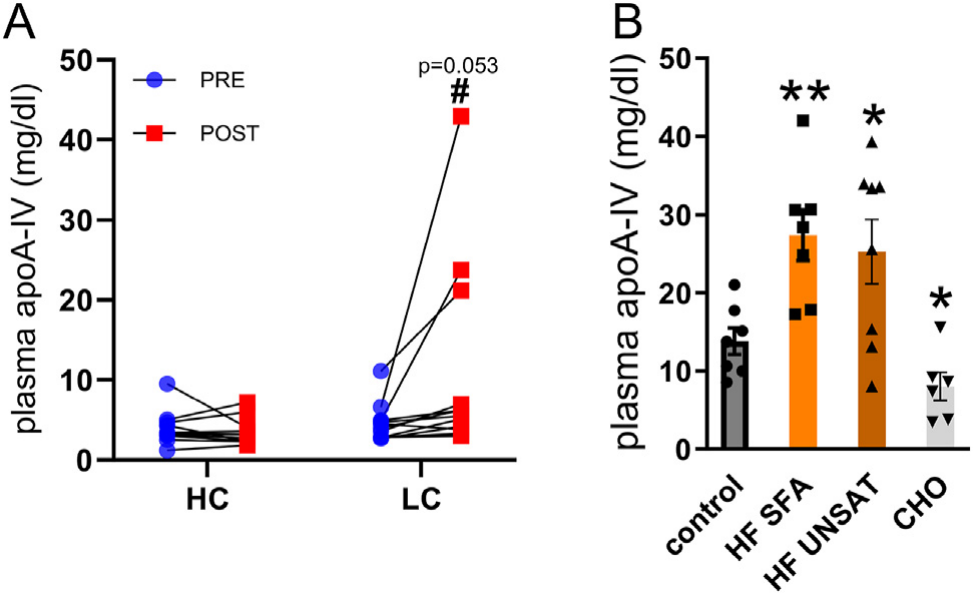
**Dietary regulation of fasting plasma apoA-IV concentration in human individuals.** Fasting plasma concentration of apoA-IV was measured in the overnight fasted state following either A) five days eucaloric intake of high-fat low-carbohydrate (LC) diet (70E% fat, 14E% carbohydrate) or low-fat high-carbohydrate (HC) diet (14E% fat, 70E% carbohydrate), and B) three days intake of a high-fat unsaturated diet (HFUNSAT) (78E% fat, 10 E% carbohydrate, 12 E% protein), a high-fat saturated diet (HFSAT) (83E% fat, 9E% carbohydrate, 12E% protein), or a high-carbohydrate diet (CHO) (80 E% carbohydrate, 11 E% protein, 9 E% fat) during 75% caloric excess, compared with a eucaloric control diet (24E% fat, 62E% carbohydrate, 14E% protein). Data are means +/− SEM. Two-way RM ANOVA was applied in A, with Sidak’s multiple comparisons post-hoc test when interaction was detected by ANOVA, paired t-test was applied in B. ∗*p* < 0.05, ∗∗*p* < 0.01 difference between the respective diet intervention and control diet/pre-intervention. #*p* < 0.05 difference between post-LC and post-HC. *n* = 11 in A, *n* = 8 in B, except for CHO group, where *n* = 5.

**Figure 2:**
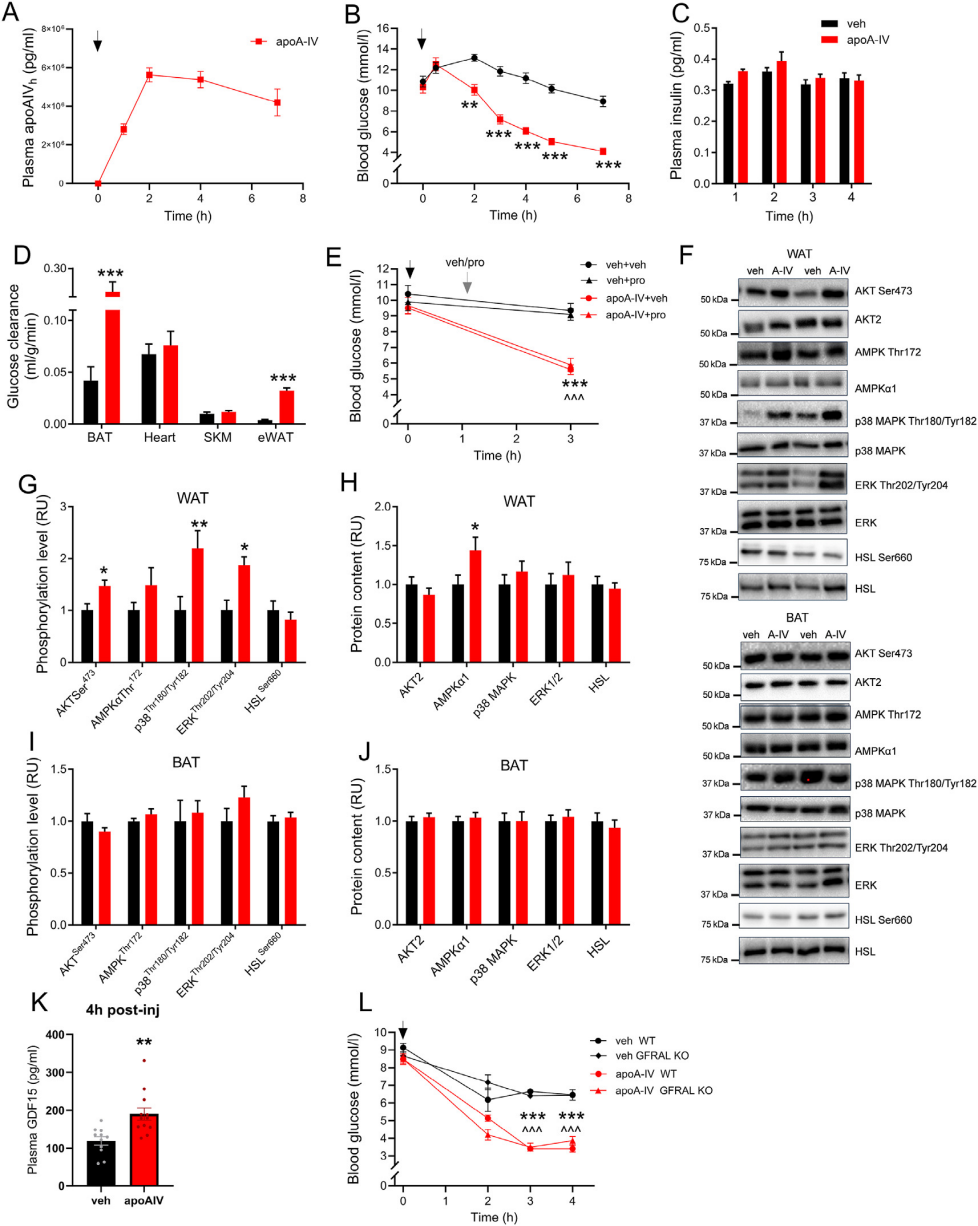
**Acute apoA-IV administration lowers fasting glucose levels in an insulin-, sympathetic activity- and GFRAL independent manner and relates to increased adipose tissue glucose disposal in mice.** Plasma concentration of human recombinant apoA-IV protein (A) and fasting blood glucose level (B) at indicated time points in mice injected at 0 h with vehicle (veh) or 1 mg/kg apolipoprotein A-IV (apoA-IV) (injection timing indicated by black arrow) (*n* = 8 in all groups). Fasting plasma insulin concentration (C) at 1 h–4 h following injection. D. Basal fasting glucose clearance into indicated tissues (BAT: brown adipose tissue, SKM: skeletal muscle, eWAT: epididymal white adipose tissue) assessed 150 min after vehicle or apoA-IV injection. E. Blood glucose before and 3 h after vehicle or apoA-IV-injection, with or without sympathetic inhibition by propranolol (pro). F. Representative blots. G. Phosphorylation level and protein content of proteins in eWAT (G–H) and BAT (I–J). K. Plasma growth differentiation factor 15 (GDF15) concentration 4 h after vehicle or apoA-IV-injection. L. Blood glucose before and 2, 3, and 4 h after vehicle or apoA-IV-injection in wildtype GDNF family receptor alpha like (GFRAL) knock-out mice. Data from western blot analyses are presented relative to the vehicle group. In all experiments, female mice were fed 60% HFD for eight weeks, and fasted prior to the experiments. Data are means ± SEM. Two-way RM ANOVA was applied in B–C, E and L, with Sidak’s post hoc test whenever interactions were detected. Unpaired t-test was applied in D, G-K. ∗*p* < 0.05, ∗∗*p* < 0.01, ∗∗∗*p* < 0.001 difference between apoA-IV and vehicle (or vehicle + vehicle in E and within genotype in L). ˆˆˆ *p* < 0.001 difference between apoA-IV and vehicle + pro. (For interpretation of the references to color in this figure legend, the reader is referred to the Web version of this article.)

**Figure 3:**
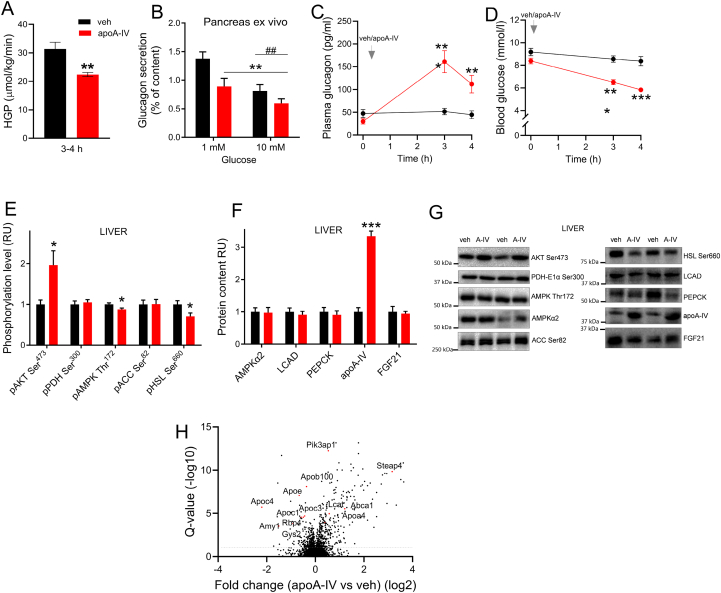
**Acute apoA-IV administration leads to suppression of basal hepatic glucose production and *ex vivo* pancreatic glucagon secretion, while changing liver protein abundance in direction of plasma lipoprotein clearance.** A. Fasting hepatic glucose production, measured over 75 min by continuous 3-^3^H-glucose infusion in anesthetized mice, i.e., at 150 min–225 min following vehicle (vehicle, *n* = 7) or 1 mg/kg apolipoprotein A-IV (apoA-IV, *n* = 9) administration. B. Glucagon secretion of isolated islets incubated with vehicle or apoA-IV (*n* = 6). Plasma glucagon (C) and blood glucose (D) concentration after injection with vehicle or apoA-IV (*n* = 10). E + F. Phosphorylation level and protein content of liver proteins obtained in the fasted state 4 h following vehicle or apoA-IV treatment (*n* = 9). G. Representative western blot of liver samples. H. Volcano plot illustrating changes in the liver proteome with apoA-IV compared with vehicle administration, with liver tissue obtained 6 h post-injection. The significance level was set at *p* < 0.05. Mice were in all experiments fed 60% HFD for 8 weeks and fasted prior to the experiments. Data are means +/− SEM. Two-way RM ANOVA was applied in B-D, with Sidak’s post hoc test whenever interactions were detected by ANOVA. Unpaired t-test was applied in A, E-F. ∗*p* < 0.05, ∗∗*p* < 0.01 ∗∗∗*p* < 0.001 difference between apoA-IV and vehicle (or main effect of apoA-IV). ##*p* < 0.01 effect of glucose concentration.

**Figure 4:**
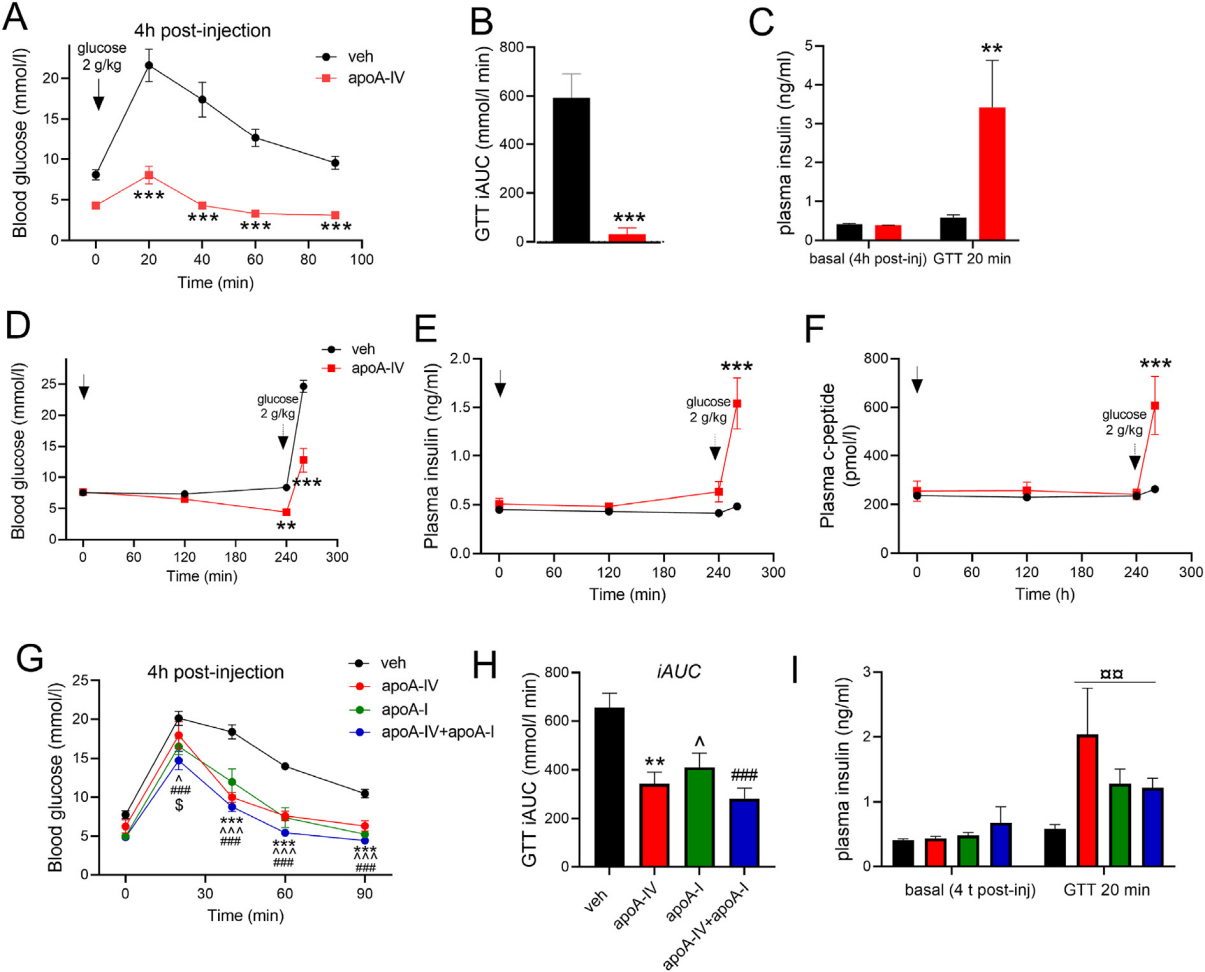
**Acute administration of apoA-IV increases glucose tolerance in obese mice, associated with increased glucose-stimulated but not basal fasting insulin secretion.** Blood glucose (A), blood glucose incremental area under curve (iAUC) (B) and plasma insulin concentration (C) in mice subjected to an intraperitoneal glucose tolerance test (GTT) (2 g glucose/kg BW) 4 h after injection with vehicle (veh) or apolipoprotein A-IV (apoA-IV; 1.0 mg/kg apoA-IV). Blood glucose (D), plasma insulin (E) and plasma C-peptide (F) concentrations measured for 4 h at fasting conditions and 20 min after intraperitoneal administration of 2 g/kg glucose. The effect of 0.5 mg/kg BW apoA-IV, 5 mg/kg BW apoA-I, and co-administration of 0.5 mg/kg apoA-IV + 5 mg/kg apoA-I were compared in G-I, showing blood glucose (G), blood glucose incremental area under curve (iAUC) (H) and plasma insulin concentration (I) before and during a 2 g glucose/kg intraperitoneal GTT 4 h after injection. Mice were in all experiments fed 60% HFD for 16 weeks, and fasted prior to the experiments. *n* = 8 in A-F, *n* = 6 in G-I. Data are means +/− SEM. Two-way RM ANOVA was applied in A, C, D-I, with Sidak’s multiple comparisons post-hoc test when interactions were detected by ANOVA. Unpaired t-test was applied in B. ∗∗*p* < 0.01, ∗∗∗*p* < 0.001 are differences between apoA-IV and vehicle. ˆ *p* < 0.05, ˆˆˆ*p* < 0.001 apoA-I versus vehicle. ##*p* < 0.01, ###*p* < 0.001 apoA-IV + apoA-I versus vehicle. $*p* < 0.05 apoA-IV versus apoA-IV + apoA-I.

#### 2.3.2 Recombinant apolipoprotein A-I

Human apolipoprotein A-I (apoA-I) containing a His-tag at the N-terminus was expressed in *E. coli* and purified as previously described [30]. ApoA-I was administrated by intraperitoneal injection at 0.5 mg/kg body weight (BW) alone or together with apoA-IV, diluted in 10 μl saline per gram BW.

### 2.4 Mouse experiments

#### 2.4.1 Measurements at fasting conditions after apoA-IV administration

*Plasma for metabolites and hormones* (Figure 2, Figure 3, Figure 4, Figure 6). In female C57BL/6JRj mice aged 14–20 weeks, after administration of vehicle or apoA-IV, blood was sampled from mixed tail blood at basal (0), 0.5, 1, 2, 3, 4, 5 and/or 7 h post-injection, as indicated. After centrifugation, plasma aliquots were stored at −30 °C for the specified analyses.

**Figure 5:**
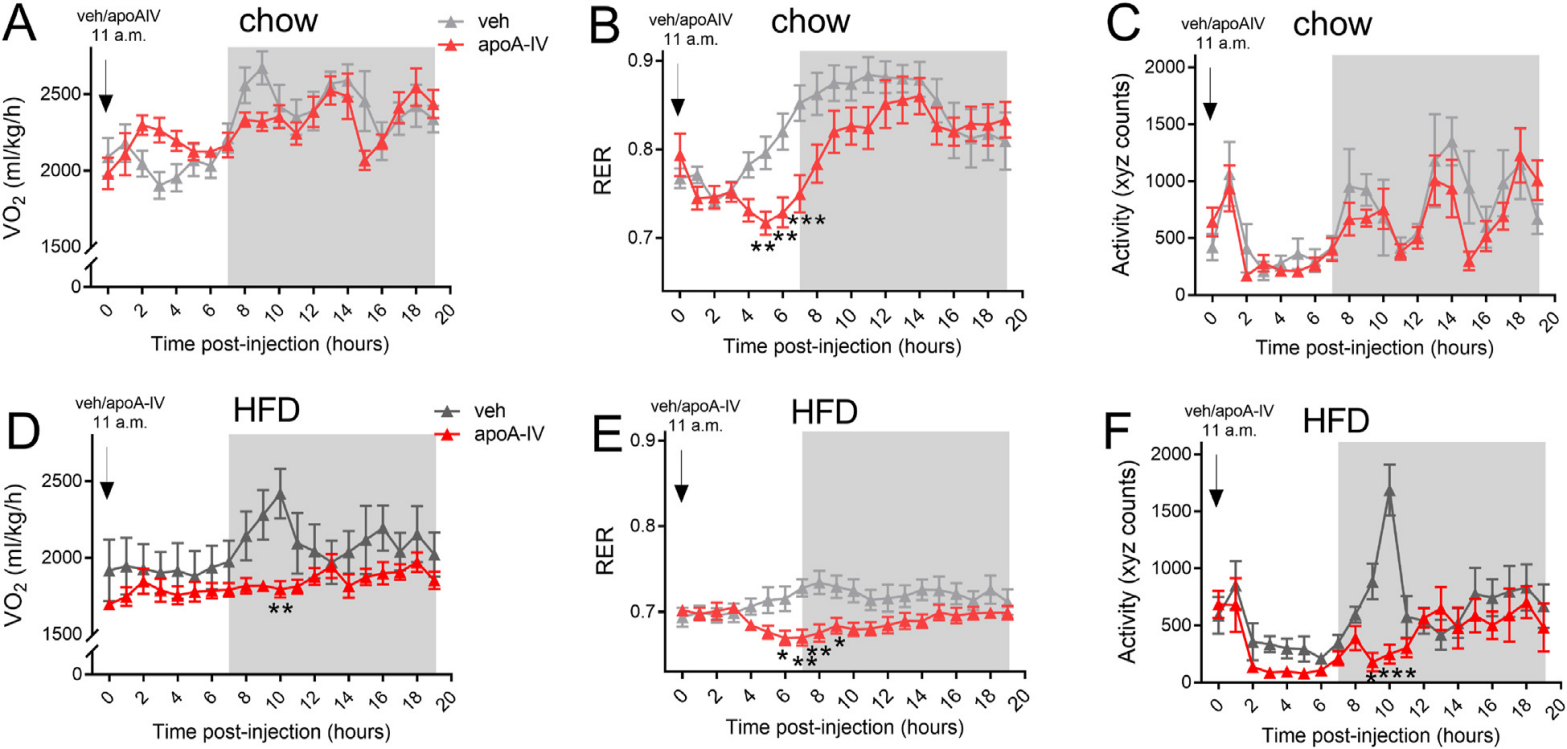
**Acute administration of apoA-IV increases whole-body fatty acid oxidation.** Metabolic chamber oxygen uptake (VO_2_) (A + D), respiratory exchange ratio (RER) (B + E) and activity level (C + F) in lean chow-fed mice (A–C) and in obese high-fat diet fed mice (D–F) measured for 20 h post-injection in the light and dark cycle after injection with vehicle (veh) or 1 mg/kg apolipoprotein A-IV (apoA-IV) at 11 a.m. (*n* = 8 in all groups). Mice had ad libitum access to their respective diets. Data are means +/− SEM. Two-way RM ANOVA was applied in A-F, with Sidak’s post hoc test when interactions were detected by ANOVA. ∗*p* < 0.05, ∗∗*p* < 0.01 difference between vehicle and apoA-IV treated mice.

**Figure 6:**
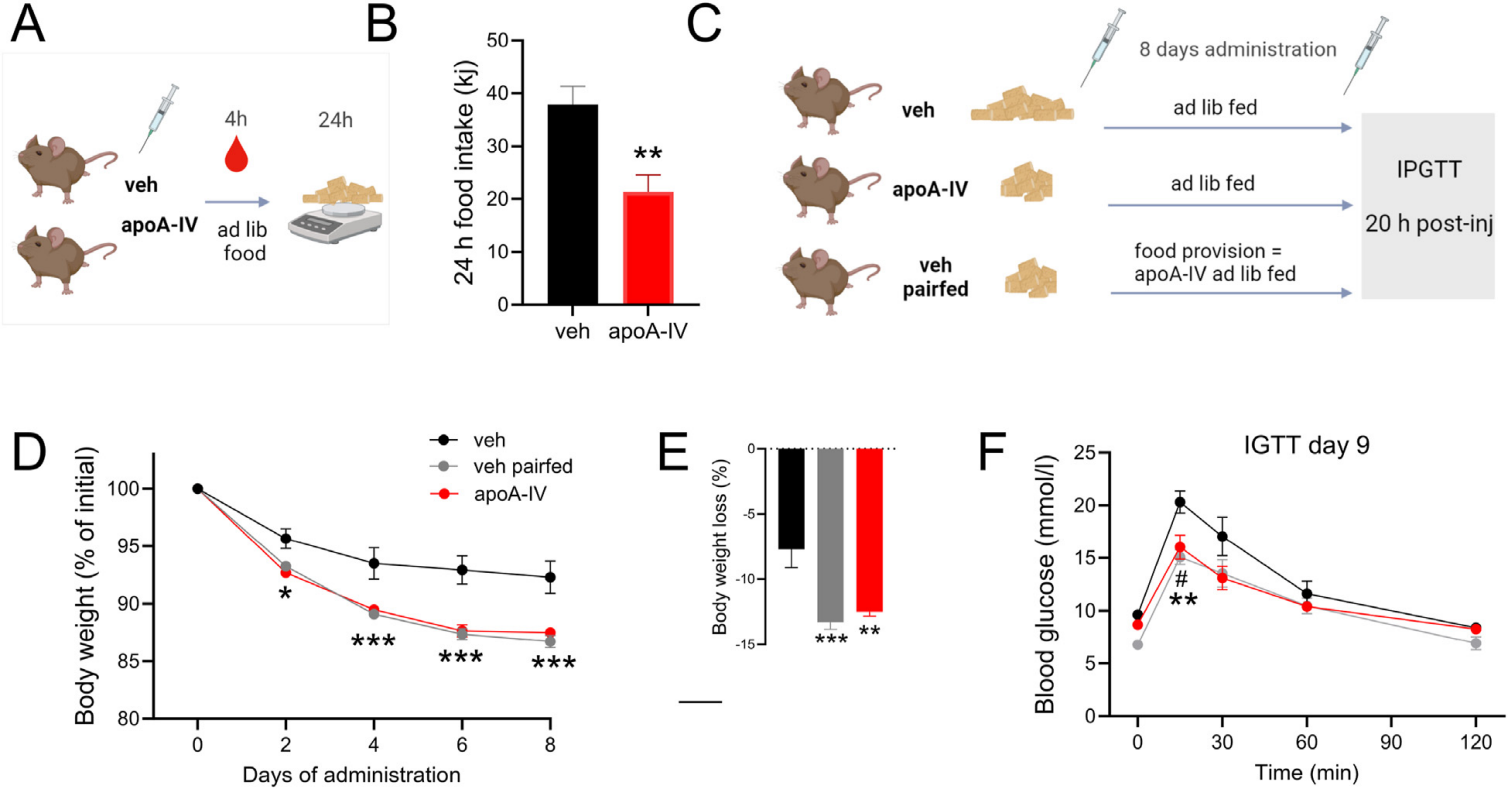
**Acute administration of apoA-IV lowers food intake, and prolonged daily administration leads to weight loss and increased glucose tolerance in obese mice.** A. Female mice fed 60% HFD were injected with vehicle (veh) or 1 mg/kg apolipoprotein A-IV (apoA-IV) and cumulative food intake was assessed after 24 h (B). Three groups of mice were either ad libitum fed the HFD and vehicle-treated (vehicle), ad libitum fed HFD and apoA-IV treated (apoA-IV) and vehicle-treated while pair fed the HFD to the energy intake of the apoA-IV-treated mice (vehicle pair fed) for eight days, with daily injection at 12 a.m. (C). Body weight is shown as % of initial body weight (D). The total %weight lost is shown in E. At day nine, mice were fasted in the morning and subjected to an intraperitoneal glucose tolerance test (2 g glucose/kg BW) with blood glucose levels shown in F. *n* = 8 in all groups. Data are means +/− SEM. Unpaired t-test was applied in B. Two-way RM ANOVA was applied in D + F, with Sidak’s post hoc test when interactions were detected by ANOVA. One-way ANOVA was applied in E. ∗*p* < 0.05, ∗∗*p* < 0.01, ∗∗∗*p* < 0.001 difference between vehicle and apoA-IV. #*p* < 0.05 difference between vehicle and vehicle pair fed mice.

*In vivo tissue-specific glucose clearance* (Figure 2). In female C57BL/6JRj mice aged 14–20 weeks, vehicle or apoA-IV were administrated, and at 150 min post-injection, the time point close to the steepest blood glucose decrement (Figure 2B), ^3^H-2-deoxyglucose (^3^H-2DG) (0.6 μCi/g BW) was intraperitoneally injected. Blood glucose was measured, and blood obtained for ^3^H-enrichment analysis at 0, 15, 30 and 45 min after ^3^H-2-DG administration (∼3.5 h post-injection). Mice were sacrificed by cervical dislocation at 45 min, and tissues were quickly excised (gastrocnemius muscle, heart, epididymal white adipose tissue (eWAT), and brown adipose tissue (BAT)) and snap-frozen in liquid nitrogen. Tissues were analyzed for ^3^H-2DG uptake, and phosphor-regulation/expression of proteins of interest.

*Sympathetic blockade* (Figure 2E). In female C57BL/6JRj mice aged 14–20 weeks, the glucose lowering effect of apoA-IV was also investigated during inhibition of sympathetic nervous activity 1 h after apoA-IV administration by blocking catecholamine action with propranolol (#P0884, Sigma, US), by injection at 5 mg/kg BW.

*GFRAL KO mice* (Figure 2L). The glucose lowering effect of apoA-IV was also investigated in mice lacking the GDF15 receptor, GFRAL. After administration of vehicle or apoA-IV, blood was sampled from mixed tail blood at basal (0), 2, 3, and 4 h post-injection in GFRAL KO and WT littermates, as indicated.

*Fasting hepatic glucose production* (Figure 3A). In female C57BL/6JRj mice aged 14–20 weeks, endogenous glucose rate of appearance was evaluated 150 min after vehicle or apoA-IV treatment in 2 h fasted mice. Mice were anesthetized with an intraperitoneal injection of 11 μl/g BW of Fentanyl (0.05 mg/ml, Dechra, DK), Midazolam (5 mg/ml, Accord Healthcare, UK), and Acepromazine (10 mg/ml, Pharmaxim, SE), and placed on a heating pad. A polyethylene cannula (PE50, Intramedic, US) was inserted into a jugular vein for administration of anesthetics and labeled glucose. Anesthesia was maintained by constant infusion of the anesthetics (0.03 μl/g). After surgery, a 75 min continuous infusion (0.83 μl/min, 1.2 μCi/h) of D-[3-^3^H]-glucose (Perkin Elmer, US) was administrated. Blood was sampled from the tail at 75 min for determination of plasma glucose and plasma ^3^H enrichment.

#### 2.4.2 Ex vivo glucagon secretion (Figure 3B)

Female C57BL/6JRj mice aged 14–20 weeks on a chow diet were euthanized by cervical dislocation. Liberase injection (Roche, CH) was used to inflate and digest the pancreas. The digested pancreata were washed with Hanks buffered saline solution (Sigma, US) + 2% BSA before hand-picking into RPMI 1640 medium (Thermo Fisher Scientific, US), supplemented with 10% FBS, 100 U/ml penicillin, 100 μg/ml streptomycin and 7 mM glucose.

Glucagon secretion was measured from groups of 10 islets per replicate. Islets were pre-incubated for 1 h at 37 °C, 5% CO2 at 1 mM glucose in Krebs Ringer Buffer (KRB) at pH 7.4 containing a physiological mix and concentration (0.36 mM) of non-esterified fatty acids. Islets were stimulated sequentially with KRB+1 mM glucose, followed by KRB+10 mM glucose, in the presence or absence of 10 μg ApoA-IV as indicated. Following secretion, islets were harvested in acid ethanol (23.5% acetic acid, 1.5% hydrochloric acid in ethanol) to quantify total islet glucagon content.

Glucagon concentrations in islet supernatants and islets were analyzed using the U-PLEX Metabolic Group 1 Multiplex Assay Kit (Meso Scale Diagnostics, US).

#### 2.4.3 Experiments with glucose exposure

*Glucose tolerance* (Figure 4A–C). In female C57BL/6JRj mice aged 14–20 weeks, 4 h after apoA-IV administration, a glucose tolerance test (GTT) was commenced in the fasted state with an intraperitoneal injection of 2 g glucose/kg BW and blood glucose determinations in mixed tail blood at 0, 20-, 40-, 60-, and 90-min. Plasma was obtained for insulin analysis at 0 and 20 min.

*Glucose-induced insulin secretion* (Figure 4D–F). In female C57BL/6JRj mice aged 14–20 weeks, vehicle or apoA-IV was administrated, and fasting blood glucose measured at 2 h and 4 h post-injection. At 4 h after apoA-IV administration, 2 g/kg glucose was intraperitoneally injected, with tail blood obtained prior to and 20 min after glucose injection for plasma insulin and c-peptide analyses.

#### 2.4.4 Experiments with oral lipid exposure (Figure 5G–J)

In female C57BL/6JRj mice aged 14–20 weeks, vehicle or apoA-IV was administered, and at 90 min mice were orally gavaged with 150 μl of corn oil, to mimic acute oral fat intake. Mixed tail blood was obtained prior to vehicle/apoA-IV injection (−1.5h), prior to lipid gavage (0) and 1, 2 and 4 h post-gavage, for measurement of plasma TG and FA, and blood ketone body and glucose levels.

#### 2.4.5 Indirect calorimetry measurements in metabolic cages (Figure 5A–F)

Female C57BL/6JRj mice aged 14–20 weeks were acclimatized for three days to individual airtight calorimetric cages connected to an indirect calorimetric system (Phenomaster/LabMaster system; TSE Systems, GE). Vehicle or apoA-IV was administrated at 11 a.m. (7 h prior to the dark cycle), and O_2_ consumption (VO_2_), CO_2_ production (VCO_2_), and physical activity level (laser beam breaks) were recorded for a 20 h period while the mice had ad libitum access to either chow or 60% HFD, as indicated. Mice were maintained on their regular 12h:12 h light:dark cycle.

#### 2.4.6 Food intake and prolonged administration of apolipoprotein A-IV (Figure 6)

Female C57BL/6JRj mice aged 14–20 weeks were fed 60% HFD for 8 weeks prior to the experiment. Mice were single-housed and received daily injections of either vehicle or apoA-IV, according to intervention group: a) vehicle, ad libitum fed, b) apoA-IV injection, ad libitum fed, and c) vehicle, pair fed the amount of HFD ingested by the apoA-IV group. Following the first vehicle or apoA-IV administration, food intake was measured the first 24 h of vehicle- and apoA-IV-treated mice (Figure 6A). After eight days of injections, a glucose tolerance test (GTT) (2 g/kg BW) was performed the following morning (20 h after the last administration of vehicle/apoAIV).

### 2.5 Analyses

*Blood and plasma analyses.* Concentrations of human apoA-IV in human and mouse plasma were measured by ELISA (#ab214567, Abcam, UK). In mice, blood glucose was measured in mixed tail blood with a glucometer (Contour XT, Bayer, CH). Plasma insulin (Mercodia, US) and C-peptide (Alpco, US) concentrations were measured by ELISA and glucagon concentrations were analyzed using the U-PLEX Metabolic Group 1 Multiplex Assay Kit (Meso Scale Diagnostics, US). Plasma GDF-15 were measured by ELISA (Mouse/Rat GDF-15, R&D Systems, US). The concentrations of plasma FA (NEFA C kit, Wako Chemicals, GE), and triacylglycerol (TG) (GPO-PAP kit, Roche Diagnostics, DE) were measured colorimetrically on a Pentra C400 analyzer (Horiba, JA). Blood ketone body levels were measured in mixed tail blood by a ketometer (Freestyle, Precision β-ketone, Abbott, US). Blood and plasma ^3^H enrichment was measured by scintillation counting.

*Western Blot Analyses.* Tissues were homogenized in ice-cold homogenization buffer, rotated end-over-end for 1h, and lysates obtained after centrifugation at 12,000 g at 4 °C. Lysates were subjected to protein determination, diluted to the same protein concentration, and subjected to SDS-PAGE and immunoblotting [31]. The primary antibodies are described in table S1. Bands were visualized using a Bio-Rad ChemicDoc MP Imaging System (Bio-Rad, US). Membranes were Coomassie-stained to check for equal loading and transfer.

*Liver protein mass spectrometry*. Liver tissue was immediately snap frozen using freeze clamps. Samples were lysed and prepared for LC/MS–MS as previously described [32]. In short, tissue was lysed in 4% sodium deoxycholate (SDC) and 100 mM tris–HCl (pH 7.5) at RT. Lysates were heated to 95 °C for 10 min, sonicated and spun at 18,000 g for 10 min at RT. Protein concentration was determined using a BCA total protein assay (Pierce). Proteins were reduced using 10 mM TCEP and alkylated using 40 mM chloroacetamide simultaneously at 95 °C for 10 min. Samples were diluted to 1% SDC using water and digested for 16 h using MS-grade trypsin at a ratio of 1:50 trypsin to protein. Peptide samples were purified using SDB-RPS stagetip cleanup. For the LC-MS/MS, peptides were injected onto a fused silica analytical column using a Dionex U3000 RSLCnano, coupled to a nanospray ESI source. Peptides were resolved over a gradient from 7% to 35% acetonitrile over 120 min with a flow rate of 300 nL/min. Peptide ionization by electrospray occurred at 2.3 kV. A Fusion Lumos mass spectrometer (ThermoFisher) with data-dependent acquisition and HCD fragmentation was used for MS/MS analysis. The mass spectrometry proteomics data have been deposited to the ProteomeXchange Consortium via the PRIDE partner repository with the dataset identifier PXD055918 (Username: reviewer_pxd055918@ebi.ac.uk, Password: 0VRnMxph8kRx). RAW data were analysed using the quantitative proteomics software MaxQuant (version 1.6.3.4). This version of MaxQuant includes an integrated search engine, Andromeda. Peptide and protein level identification were both set to a false discovery rate of 1% using a target-decoy based strategy. The database supplied to the search engine for peptide identifications contained both the mouse UniProt database and the MaxQuant contaminants database. Mass tolerance was set to 4.5 ppm for precursor ions and MS/MS mass tolerance was 20 ppm. Enzyme specificity was set to trypsin (cleavage C-terminal to Lys and Arg) with a maximum of 2 missed cleavages permitted. Deamidation of Asn and Gln, oxidation of Met, pyro-Glu (with peptide N-term Gln) and protein N-terminal acetylation were set as variable modifications. N-ethylmaleimide on Cys was searched as a fixed modification. We used the MaxLFQ algorithm for label-free quantitation, integrated into the MaxQuant environment.

*Liver glycogen.* Liver glycogen content was determined fluorometrically as glycosyl units after acidic hydrolysis [33].

*Liver TG content*. Liver TG content was measured in 20 mg tissue photometrically as described previously [6].

*Tissue*^*3*^*H-2DG uptake*: Blood ^3^H activity was measured in 5 μl blood by scintillation counting. 10–40 mg of each tissue was used to determine the accumulation of phosphorylated ^3^H-2-DG (^3^H-2-DG-6-P) by the precipitation method [34]. Glucose clearance was calculated by dividing tissue ^3^H-2-DG-6-P DPM per 45 min by average blood ^3^H-2-DG DMP.

### 2.6 Statistical analyses

All data are expressed as means ± SEM of one experiment. The statistical analyses performed are described in each figure legend. Differences between vehicle and apoA-IV administration were analyzed by Students unpaired t-test, one-way ANOVA, or two-way ANOVA with or without repeated measurements as appropriate. Šidák was used for post hoc testing. Statistical significance was defined as *p* < 0.05. Statistical analyses were performed in GraphPad PRISM 9 (GraphPad, US). Liver proteomics data was analyzed in R. Fold changes were calculated on group basis, based on median values. Statistical significance was determined using a two-way ANOVA. Statistical outputs were corrected for multiple hypothesis testing using the Benjamini-Hochberg correction, with significance being set at *p* < 0.05 at an FDR of 5%.

## 3 Results

### 3.1 High-fat and low-carbohydrate intake increase fasting apoA-IV levels in human individuals

Previously, plasma proteomics analysis revealed that six weeks eucaloric intake of both a saturated and unsaturated high-fat diets led to increases in fasting plasma apoA-IV in mice (2.2-fold) and healthy humans (1.4-fold) [6]. We therefore proceeded to investigate this regulation further. Here, a 151% increase in fasting plasma apoA-IV concentration was observed in healthy men with overweight or obesity after five days intake of a eucaloric high-fat (71E%), low-carbohydrate diet, whereas no changes were observed after 5 days intake of low-fat (14E%), high-carbohydrate diet (study 1; Figure 1A). Furthermore, in lean, healthy men subjected to three days overfeeding (+75% energy) with fat (78-83E% fat), a similar increase in fasting plasma apoA-IV concentration was found after both a saturated (+98%) and a unsaturated (+83%) high-fat diet, while a similar overfeeding with carbohydrates (80E% carbohydrate) lowered fasting plasma apoA-IV concentrations (study 2; Figure 1B). Circulating apoA-IV levels are thus increased in the overnight-fasting state upon short-term high-fat intake, independent of dietary fat type and in both lean men and men with overweight or obesity.

### 3.2 Acute apoA-IV administration lowers fasting blood glucose in an insulin-independent manner

To investigate the physiological and metabolic effects of apoA-IV, we produced human recombinant apoA-IV protein and verified it for purity (fig S1) and functionality (i.e., lipid binding and cholesterol efflux capacity, fig. S2). Intraperitoneal administration of human recombinant apoA-IV protein to HFD-induced obese female mice gave rise to a peak in the plasma concentration of the apoA-IV human protein at 2 h following injection, which remained elevated and still above the half peak concentration 7 h after injection (Figure 2A). In response to apoA-IV, fasting blood glucose was lowered 2 h after administration and onwards compared with vehicle, and blood glucose at 7 h averaged 4.1 ± 0.3 and 8.9 ± 0.5 mmol/l in apoA-IV- and vehicle-treated mice, respectively (Figure 2B). The lowering of blood glucose was not associated with a higher plasma insulin concentrations measured 1 h, 2 h, and 4 h after injection of ApoA-IV vs. control (Figure 2C). ApoA-IV induced a similar lowering of fasting blood glucose in male and female mice, as well as in chow- and high-fat, high-sucrose fed mice (fig. S3). The lowering of fasting blood glucose levels was obtained concomitantly with an increased basal glucose clearance into BAT (5.0-fold) and eWAT (8.8-fold) compared with vehicle (Figure 2D). Inhibition of sympathetic activity by propranolol did not alter apoA-IV's blood glucose lowering effect (Figure 2E), therefore, the apoA-IV-induced lowering of fasting blood glucose levels appeared independent of beta-adrenergic regulation. The increased glucose clearance into eWAT following apoA-IV administration was obtained together with 47% increased phosphorylation of AKT Ser^473^, despite the unchanged fasting plasma insulin levels. Also, apoA-IV induced an increased phosphorylation of p38 mitogen-activated protein kinase (p38 MAPK) and extracellular-signal regulated kinase (ERK), and 44% increased protein content of AMP-activated kinase α1 (AMPKα1), measured 3 h post-injection (Figure 2G+H). In BAT, phosphorylation level or protein content of molecules involved in insulin-, MAPK-, ERK- and AMPK-signaling were not affected by apoA-IV administration (Figure 2I+J). Plasma GDF15 concentration was 60% higher compared with vehicle at 4 h after administration of apoA-IV (Figure 2K). However, GDF15 seemed not to mediate the blood-glucose lowering effect of apoA-IV, since WT and GDF15-receptor KO mice lowered blood glucose levels similarly in response to apoA-IV administration (Figure 2L).

### 3.3 Acute apoA-IV administration lowers hepatic glucose production

To investigate whether the glucose lowering effect of apoA-IV was related to lowering of endogenous glucose production in addition to enhanced peripheral glucose disposal, basal endogenous glucose production in the fasted state was measured in anesthetized mice at 3 h–4 h after vehicle or apoA-IV administration. This revealed a 29% lower basal endogenous glucose production in apoA-IV treated mice (Figure 3A). Since glucagon is a major activator of hepatic glucose production, we investigated the role of apoA-IV in regulation of glucagon secretion. Interestingly, *ex vivo* incubation of isolated islets revealed inhibition of glucagon secretion by apoA-IV with the presence of 1 mM as well as 10 mM glucose in the media (Figure 3B), suggesting that apoA-IV *per se* lowers glucagon secretion. However, when plasma glucagon was assessed *in vivo*, glucagon concentrations were found to be elevated in apoA-IV treated mice (Figure 3C), likely related to the lower blood glucose compared with vehicle or an effect of ApoA-IV on glucagon clearance in the liver (Figure 3D). Thus, while apoA-IV could hypothetically have induced blood glucose reduction by inhibition of glucagon-stimulated hepatic glucose output, an apoA-IV suppression of circulating glucagon levels could not be verified during these conditions of reduced blood glucose. Western blot analysis of hepatic proteins at 4 h revealed increased liver AKT Ser^473^ phosphorylation in apoA-IV-treated mice (Figure 3E+G) as a potential contributing mechanism for the reduced liver glucose production, but with circulating insulin concentrations being unchanged (Figure 2C). Protein content of the rate-limiting enzyme in gluconeogenesis, phosphoenolpyruvate carboxykinase (PEPCK), was however unchanged (Figure 3F+G). Proteomics was condutcted to obtain a global screen of pathways involved in apoA-IV effects (supplementary table 2.) The proteomics analysis of liver tissue obtained at 6 h after vehicle or apoA-IV administration revealed no overall changes in proteins related to gluconeogenesis. However, altered enrichment of protein abundances related to plasma lipoprotein clearance was found by the Msig Database. More specifically, lower protein abundance was noted for apoB-100 (−1.3-fold), apoE (−1.6-fold), apoC-I (−1.5-fold), apoC-III (−1.4-fold), apoC-IV (−4.6-fold) (Figure 3H) compared with vehicle within the 6 h timeframe, which together could indicate an increased secretion of VLDL and HDL lipoproteins from the liver, carrying these apolipoproteins. An increased abundance was obtained for ATP-binding cassette transporter 1 (ABCA1) (2.3-fold) and lecithin-cholesterol acyltransferase (LCAT) (1.3-fold), proteins important to reverse cholesterol transport. Within glucose metabolism, lower abundance of glycogen synthase (GYS2) (−1.4-fold) and alpha-amylase (AMY1) (−2.8-fold) were obtained, which could indicate reduced glycogen synthesis. Interestingly, liver protein abundance of retinol-binding protein 4 (RBP4), described to be associated with insulin resistance and adipose tissue inflammation (Norseen et al., 2012), was lowered with apoA-IV administration (−1.9-fold), thus pointing to apoA-IV being a novel regulator of RBP4. Moreover, a marked increase in STEAP4 was obtained (8.9 -old), which is of interest as STEAP4 protein is found to be reduced in the liver of individuals with NAFLD, and overexpression in mice ameliorates liver steatosis and insulin resistance [35].

### 3.4 Acute administration of apoA-IV increases glucose tolerance and potentiates glucose-stimulated insulin secretion

Besides the regulation of glycemia at basal conditions, glucose tolerance after an interperitoneal glucose tolerance test was assessed in HFD-induced obese mice at 4 h following apoA-IV administration. Peak blood glucose averaged 21.6 ± 2 and 8.1 ± 1.1 mmol/l in vehicle- and apoA-IV-treated mice, respectively, at 30 min (Figure 4A), and the glucose incremental area under curve (iAUC) was remarkably 95% lower in the apoA-IV group (Figure 4B).

This remarkable observation was associated with 4.7-fold higher plasma insulin concentrations 20 min after glucose injection in apoA-IV compared with vehicle (3.4 ± 1.2 ng/ml versus 0.6 ± 0.1 ng/ml), while basal plasma insulin levels were similar (Figure 4C). In a separate experiment, again conducted 4 h post-injection, an apoA-IV-mediated potentiation of glucose-stimulated insulin secretion was confirmed, as plasma insulin and C-peptide concentrations were 220% and 140% higher, respectively, at 20 min after glucose administration (Figure 4D–F).

This glucose-dependent stimulation of insulin secretion by apoA-IV mimics the action of apoA-I that also induces increased insulin dependent and -independent glucose disposal [31,36,37]. However, whereas the induction of insulin-independent glucose disposal by apoA-I is driven by increased glucose clearance into heart and skeletal muscle, here we found apoA-IV induces insulin-independent glucose uptake into adipose tissues. Thus, to investigate whether apoA-I and apoA-IV co-administration would have additive or even synergistic effects on glucose disposal, we compared glucose tolerance in mice at 4 h following matched doses of apoA-IV, apoA-I, and co-administration of apoA-IV and apoA-I (Figure 4G+H). Plasma insulin was similarly increased in the three groups, (Figure 4I), and glucose tolerance measured as iAUC of the glucose excursion curve during the GTT were lowered by 47%, 38% and 57% in the apoA-IV, apoA-I, and apoA-IV + apoA-I groups (Figure 4H), with no statistically significant additive effect in the co-administration group.

### 3.5 Acute administration of apoA-IV leads to increased fatty acid oxidation

To investigate whether apoA-IV was associated with altered substrate utilization, mice were investigated in metabolic chambers following vehicle or apoA-IV administration (early in the light cycle). The measurements revealed increased whole-body fatty acid oxidation a few hours after apoA-IV administration in both chow- and HFD-fed mice (Figure 5B+E). While the metabolic rate (oxygen uptake) was numerically increased by 12–19% in apoA-IV compared with vehicle-treated chow-fed mice, this did not reach significance (Figure 5A), and was not evident in the more obese HFD-fed mice (Figure 5D). The oxygen uptake was lower at the onset of the dark cycle after apoA-IV treatment, significant only in HFD-fed mice (Figure 5D), concomitant with lower cage movement (Figure 5F), likely coupled to less food intake in apoA-IV-treated mice.

### 3.6 Prolonged administration of apoA-IV induces weight loss and improves glucose tolerance

To investigate the proposed role of apoA-IV in satiety, we measured the cumulative 24 h food intake (of HFD), which was 44% lower after mice were subjected to apoA-IV administration compared with vehicle mice (Figure 6A). To investigate the potential of chronic apoA-IV administration to lower body weight in obese mice, and to also evaluate the glucometabolic effects of chronic apoA-IV administration, obese mice were administered with apoA-IV for eight days, during which vehicle-treated mice were either ad libitum-fed or had food availability matched to the intake of apoA-IV-treated mice (pair-fed) (Figure 6B). Thus, body weight was decreased in apoA-IV-treated mice, and per design similarly in the vehicle pair-fed group (−12.5 ± 0.3% and −13.3 ± 0.5% of initial body weight) (Figure 6C+D). When glucose tolerance was assessed at day nine, 20 h following the last injection, glucose tolerance was improved, but similar, in apoA-IV and vehicle pair fed mice (peak blood glucose of 16.0 ± 1.1 and 15.1 ± 0.7 mmol/l) compared with the ad libitum vehicle group (20.3 ± 1.1 mmol/l) (Figure 6E), indicating a weight-loss related improvement of glucose tolerance by chronic apoA-IV.

## 4 Discussion

Here, we demonstrate that in both lean individuals and individuals with overweight or obesity, high-fat intake increases fasting plasma levels of apoA-IV, independent of dietary fatty acid type. To evaluate the metabolic effects of apoA-IV in a metabolically compromised setting, we administered apoA-IV to fasting obese mice revealing an acute blood glucose lowering effect. This is driven by a dual mechanism relating to both increased basal glucose uptake into white and brown adipose tissues and to inhibition of basal hepatic glucose production. These glucose metabolic effects are found together with increased whole-body fatty acid oxidation and changes in liver proteins related to altered “capacity for lipoprotein clearance” based on proteomics pathway analysis. Under conditions of glucose stimulation, improvement of glucose tolerance by apoA-IV is related to potentiation of glucose-induced insulin secretion.

### 4.1 Upregulation by dietary fat intake

We previously observed increased circulating apoA-IV abundance by proteomics analysis after intake of six weeks eucaloric saturated or unsaturated 64E% HFD in men, who were overweight or obese [6]. Here, we further show that fasting apoA-IV levels were upregulated upon increased fat (70E% to 83E%) and decreased carbohydrate intake in humans, across two independent dietary intervention studies. This is consistent with one previous study showing that fasting plasma apoA-IV levels in healthy men correlate with the level of fat intake, measured at 10E%, 25E% and 50E% fat [38]. The increase in fasting concentrations could be indicative of an increased hepatic protein abundance and secretion of apoA-IV, a mechanism which is likely in contrast to the intestinal apoA-IV induction with acute fat intake, and awaits to be investigated further.

### 4.2 The glucose lowering action of apoA-IV

To evaluate the role of increased circulating levels of apoA-IV, we administered apoA-IV to fasted obese mice and observed lowering of blood glucose levels accompanied by increased basal glucose uptake by 5- and 9-fold into brown and white adipose tissue, respectively. This observation adds to a previous report in which acute administration of recombinant mouse apoA-IV protein was shown to increase *glucose-stimulated* but not basal glucose uptake in BAT and WAT of lean chow-fed mice [39]. In the present study, AKT Ser^473^ phosphorylation was increased concomitantly with the increased glucose uptake in WAT, notably independent of any increase in circulating insulin levels. In line with this, it has been demonstrated *in vitro* in 3T3L1 adipocytes that apoA-IV increased basal as well as insulin-stimulated glucose uptake in a PI3K-dependent manner. This effect was associated with activation of the low-density lipoprotein receptor-related protein 1 (LRP1) [40], which is the only known receptor for apoA-IV. In the present *in vivo* study, apoA-IV administration also led to increased p38 MAPK phosphorylation in WAT. Potentially, this activation of MAPK signaling could lead to increased WAT adipogenesis or lipogenesis [41]. The fate of the additional glucose taken up upon apoA-IV exposure could potentially be substrate for lipogenesis, as could be the case in the physiologic condition where apoA-IV increases upon chylomicron entry into the circulation. While the increased glucose disposal into WAT seems associated with PI3K-AKT signaling, the regulatory mechanism behind the increased glucose uptake into BAT was not clear. Others have shown that mouse apoA-IV administration to the brain can lead to increased BAT thermogenesis, as evidenced by increased BAT temperature and UCP1 protein content [42] and we found a trend for increased oxygen uptake (by 16% compared with pre-injection) in the early hours after injection of apoA-IV in lean chow-fed mice. However, sympathetic blockade by propranolol did not diminish the glucose lowering effect of apoA-IV, therefore increased sympathetic activity was unlikely to mediate the observed increased adipose glucose disposal.

Administration of GDF15 was recently shown to mimic apoA-IV and improve glucose disposal into WAT and BAT and lower hepatic glucose production [28] and circulating GDF15 levels were increased 4 h after apoA-IV administration. However, the blood-glucose lowering effect of apoA-IV appeared independent of GDF15 regulation as apoA-IV lowered blood glucose levels similarly in WT and GDF15 receptor KO mice.

### 4.3 Actions of apoA-IV in the liver and pancreas

We found a suppression of hepatic/endogenous glucose production in fasting mice following apoA-IV, concomitantly with an activation of AKT signaling in the liver without concomittant increase in circulating insulin concentrations. Of relevance, one of the liver proteins with the most significant upregulation was PI3K adapter protein 1, proving activation of the PI3K-AKT pathway. The mechanisms in the reduced liver glucose output could include suppression of gluconeogenic genes, but no change in protein content of PEPCK, the rate-limiting enzyme in gluconeogenesis, was obtained in the liver following apoA-IV administration. Also, at 7 h post-injection, no changes in protein abundance related to gluconeogenesis or glycogenolysis were obtained by liver proteomics. This points to other mechanisms, likely related to altered gluconeogenic substrate availability, or reduced activity levels of glycogenolytic enzymes. The liver proteomics analysis also revealed type I interferon signaling being surprisingly upregulated with apoA-IV, which could imply some sort of a malaise/inflammatory response to injection of the recombinant protein. However, a noteworthy similar response was soothingly observed with an endogenous upregulation of apoA- IV by intermittent fasting [32,43] supporting our verification of the recombinant protein being free of endotoxins and that the observed effects of apoA- IV were reliable.

In search for potential regulators driving inhibition of hepatic glucose secretion by apoA-IV, we evaluated the glucagon response. During *in vivo* conditions, plasma glucagon levels in response to apoA-IV administration were not reduced, likely due to counter-regulatory factors related to the hypoglycemic state induced by apoA-IV. *Ex vivo* experiments were performed to circumvent this bias from other factors, and in isolated islets, apoA-IV incubation suppressed glucagon secretion, emphasizing a novel regulatory mechanism of glucagon secretion. This could imply that apoA-IV-induced inhibition of glucagon release from alpha-cells could be an initial mechanism lowering hepatic glucose output, partially driving the blood glucose lowering effect of apoA-IV. In regard to beta-cell action, we found a potent increase in glucose-stimulated insulin secretion by apoA-IV, as plasma insulin and C-peptide concentrations were higher by 220% and 131%, respectively, compared with vehicle. This likely represented the major mechanism in the apoA-IV improved glucose tolerance. The potentiation of glucose-stimulated insulin secretion has been reported before under *ex vivo* conditions in isolated pancreas [20]. The role of apoA-IV in regulating pancreatic hormone secretion is in congruence with the presence of the LRP1 receptor gene in mouse alpha and beta cells [44].

By proteomics, lower protein abundance in the liver was found for the apolipoproteins apoB-100, apoE, apoC-I, apoC-III and apoC-IV, indicative of increased production and secretion of VLDL- and HDL-lipoproteins from the liver. This supports previous suggestions of a role of apoA- IV in TG secretion from the liver, by interacting with apoB as shown in rat hepatoma cells [45] and in mice liver by infection with apoA- IV adenovirus [46]. In the present study, an increased abundance was obtained for ABCA1 and LCAT, proteins important for reverse cholesterol transport. The ability of apoA-IV to activate LCAT was early described [47], and apoA-IV now represents a potential target in treatment of atherosclerotic cardiovascular disease [16].

### 4.4 Anti-obesogenic actions of apoA-IV

With daily apoA-IV treatment, we observed lower food intake and reduced body weight. The suppression of food intake by apoA-IV has been reported to be dependent on hypothalamic activation of the melanocortin system and inhibition of neuropeptide Y [48,49].

The slight increase in oxygen consumption obtained in the metabolic chambers following apoA-IV administration in lean mice did not reach significance and collectively, a thermogenic effect of apoA-IV does thus not seem to be a major contributor to the observed weight loss of apoA-IV treatment. Chronic, daily apo-IV treatment improved glucose tolerance, which appeared related to a large extent to the apoA-IV-mediated weight loss - and not a role of apoA-IV *per se*, as a similar improvement in glucose tolerance was observed in pair-fed mice. Thus, while apoA-IV *per se* acutely improves glucose tolerance 4 h later, independently of any effect of food intake (al mice fasted during the acute studies), the effect of apoA-IV on glucose tolerance is not evident 24 h after the latest administration during a chronic intervention, except for the effect of weight loss induced by such treatment.

We found a shift in substrate utilization, as whole-body fatty acid oxidation was increased in the hours after apoA-IV administration, especially until the dark period with increased food intake. This is congruent with the concomitant increases in both circulating apoA-IV levels and whole-body fatty acid oxidation rates in response to both acute and prolonged high-fat intake and raises the possibility that apoA-IV contributes to increase fatty acid oxidation during dietary fat intake [6]. The mechanisms behind the apoA-IV-induced shift to fatty acid oxidation are not clear. While not assessed in skeletal muscle, phosphor-regulation of the metabolic master switch, PDH, in the liver was unchanged. Potentially, the effect of apoA-IV towards lowering of glucose in the circulation, along with lowering of liver glycogen content, could mediate insufficient glucose availability for the peripheral tissues forcing a higher reliance of fat for oxidation. Also, apoA-IV could likely have increased fatty acid disposal in tissues such as liver and skeletal muscle, via increased LPL activation as proposed [18].

### 4.5 Limitations

The participants in the human studies were all male adults, which may limit the generalizability of the findings to females. It has previously been shown that lipid metabolism differs between sexes [50,51], underscoring the importance of investigating whether apoA-IV is differently regulated by dietary fat in females in future studies. Along these lines, almost exclusively female mice were applied to allow for group housing, hence increasing animal welfare. Whether the metabolic response to apoA-IV is the same in male mice awaits to be investigated, although the central finding of apoA-IV lowering blood glucose was verified to be identical between male and female mice.

### 4.6 Conclusions

ApoA-IV administration improves glycemic control and hepatic lipoprotein metabolism as well as increases whole-body fatty acid oxidation and has anti-obesogenic potential in obese mice. The effects of apoA-IV is orchestrated by actions in white and brown adipose tissue, pancreas, and the liver. Fasted circulating apoA-IV levels are potently induced following few days to several weeks of increased intake of fat in humans, likely as a mechanism to ensure efficient disposal of the fat in different tissues. Our data propose that induction of apoA-IV in response to dietary fat could mediate some of the beneficial effects of dietary fat and serve to maintain insulin action, by removing glucose into adipose tissue and also suppressing glucose output from the liver, during times of increased fatty acid metabolism.

## CRediT authorship contribution statement

**Anne-Marie Lundsgaard:** Writing – review & editing, Writing – original draft, Visualization, Project administration, Methodology, Investigation, Formal analysis, Data curation, Conceptualization. **Rita Del Giudice:** Writing – review & editing, Validation, Methodology, Investigation, Formal analysis. **Josephine M. Kanta:** Writing – review & editing, Investigation. **Mark Larance:** Writing – review & editing, Resources, Methodology, Investigation. **Sarah L. Armour:** Writing – review & editing, Methodology, Investigation. **Amalie London:** Writing – review & editing, Investigation, Formal analysis. **Michael M. Richter:** Writing – review & editing, Investigation. **Nicoline R. Andersen:** Writing – review & editing, Investigation, Formal analysis. **Trine S. Nicolaisen:** Writing – review & editing, Investigation. **Christian S. Carl:** Writing – review & editing, Investigation. **Kim A. Sjøberg:** Writing – review & editing, Investigation. **Kirstine Nyvold Bojsen-Møller:** Writing – review & editing, Investigation. **Jakob G. Knudsen:** Writing – review & editing, Methodology, Investigation. **Jens O. Lagerstedt:** Writing – review & editing, Supervision, Methodology, Investigation. **Andreas M. Fritzen:** Writing – review & editing, Writing – original draft, Methodology, Investigation, Formal analysis, Data curation, Conceptualization. **Bente Kiens:** Writing – review & editing, Writing – original draft, Supervision, Resources, Investigation, Funding acquisition, Conceptualization.

## Declaration of competing interest

The authors declare the following financial interests/personal relationships which may be considered as potential competing interests: M.L. reports equipment, drugs, or supplies was provided by 10.13039/501100001774The University of Sydney. Annemarie Lundsgaard reports a relationship with Novo Nordisk that includes: employment. Jens O. Lagerstedt reports a relationship with Novo Nordisk that includes: employment. If there are other authors, they declare that they have no known competing financial interests or personal relationships that could have appeared to influence the work reported in this paper.

## Data Availability

Data will be made available on request.
