# Supplementary material for: Apolipoprotein A-IV is induced by high-fat diets and mediates positive effects on glucose and lipid metabolism
Source: Mol Metab. 2025 Mar 1;95:102119. doi: 10.1016/j.molmet.2025.102119 (PMC11938269; doi:10.1016/j.molmet.2025.102119)
Supplement: Multimedia component 1 [file mmc1.docx]

**Figure S1.** **ApoA-IV expression (left panel) and purification (right panel).** Protein expression as well as protein purity were analyzed by SDS-PAGE, followed by Coomassie staining.

**
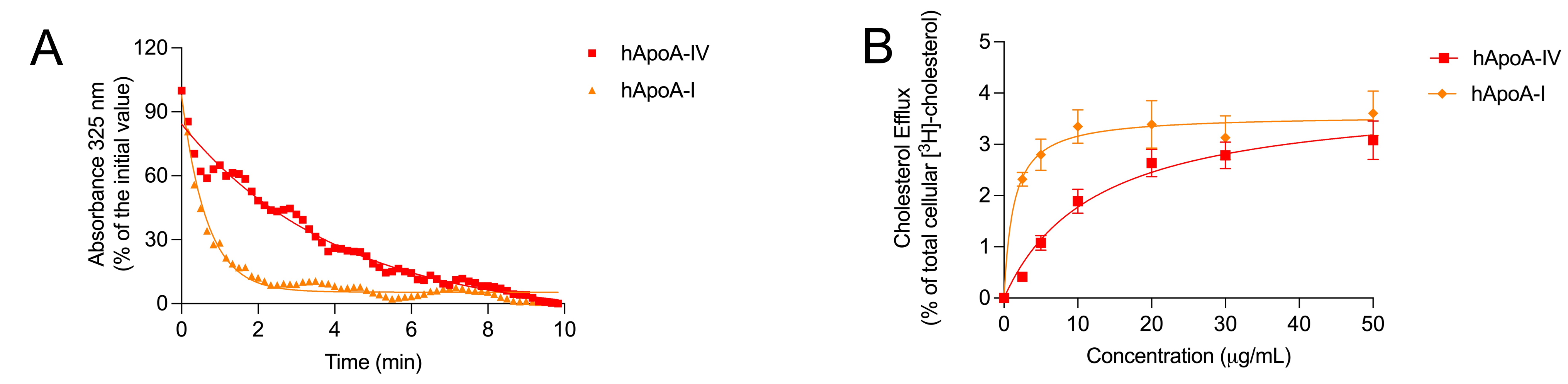
**

**Figure S2.** **ApoA-IV functionality tests.** ApoA-IV was tested *in vitro* for lipid clearance capacity (A), normalized to % of starting value, and cholesterol efflux capacity (B) together with apoA-I protein. ApoA-I lipid clearance data are obtained from a previous publication (Del Giudice et al., 2017).


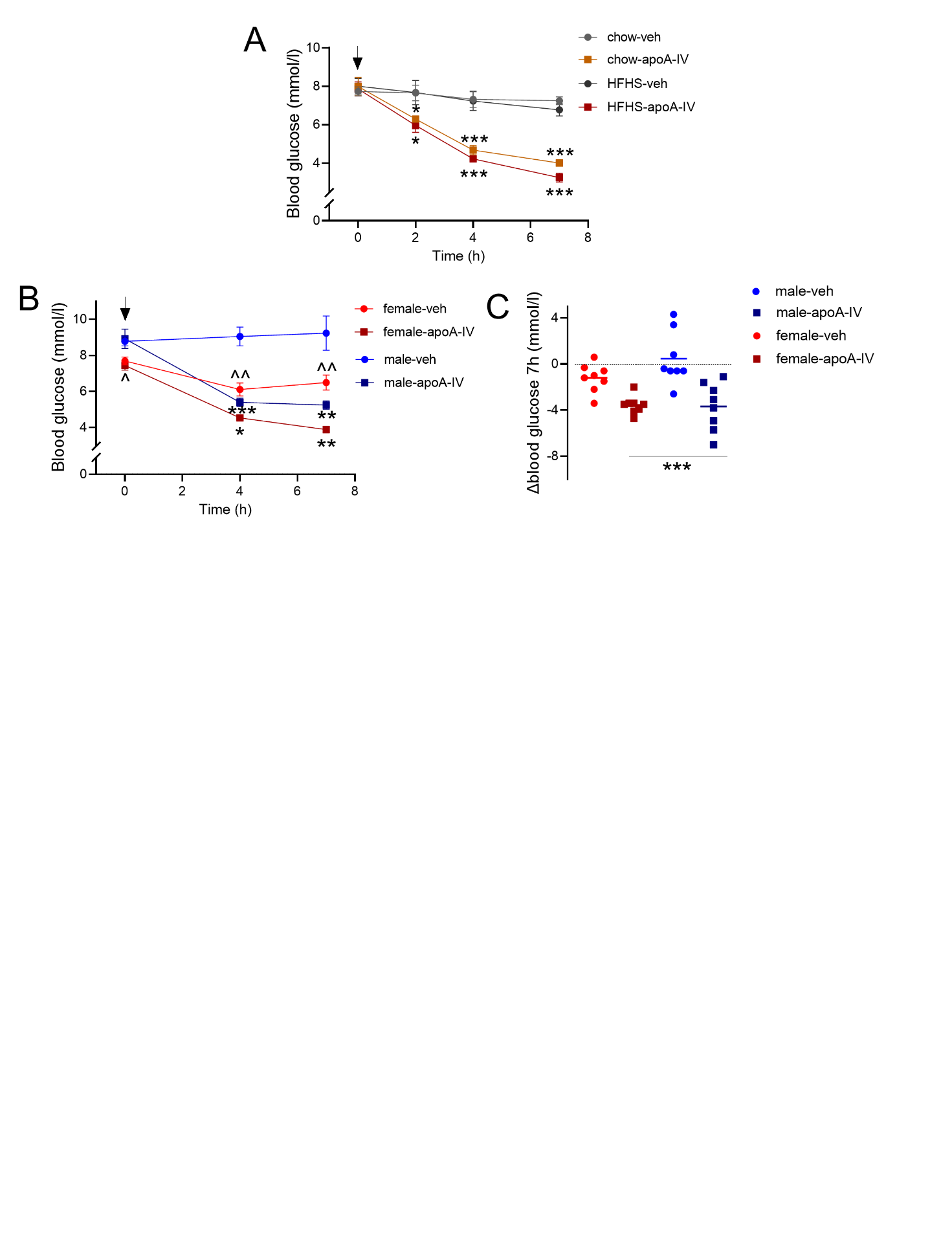


**Figure S3.** **Acute apoA-IV lowers fasting glucose in mice fed either chow or high-fat high-sucrose diet, independent of gender.** Blood glucose level at indicated time points (measured for 7h) after injection at 0h with either vehicle (vehicle) or 1 mg/kg apolipoprotein A-IV (apoA-IV) (n=8 in all groups) in female mice fed chow diet or 8 weeks high-fat high sucrose (HFHS) diet (A), and in male and female chow-fed mice (B+C). Mice were fasted prior to experiments. Data are means±SEM. Two-way RM ANOVAs were applied in A-C, with Sidak´s post hoc tests. ^^ p < 0.01 difference between male and female mice. * p<0.05, *** p < 0.001 difference between apoA-IV and vehicle (or main effect of apoA-IV).

|  |  |
| --- | --- |
| **Primary antibodies** | **Source** |
| anti-PEPCK | #61610, Santa Cruz Biotechnology, US |
| anti-AKT2 | #3063, Cell Signaling Technology, US |
| anti-AKT Ser473 | Cell Signaling Technology, US |
| anti-CD36 | #AF2519, R&D Systems, US |
| anti-PDH-E1α | in-house, Professor H. Pilegaard, Copenhagen University |
| anti-PDH-E1α Ser300 | in-house, Professor H. Pilegaard, Copenhagen University |
| anti-HSL | Donated by Dr. C Holm, Lund University, SE |
| anti-HSL Ser660 | #4126, Cell Signaling Technology, US |
| anti-AMPK𝛼2 | Donated by Dr. Hardie, Dundee University, US |
| anti-AMPK𝛼1 |  |
| anti-AMPKα Thr172 | #2531, Cell Signaling Technology, US |
| anti-FGF21 |  |
| anti-p38 Thr180/Tyr182 |  |
| anti-p38 MAPK |  |
| anti-ERK Thr202/Tyr204 |  |
| anti-ERK1/2 |  |
| anti-ACC Ser82 |  |
| anti-LCAD | #54936, Novus, |
| anti-apoA-IV | #AF8125, R&D Systems, US |
|  |  |

Table S1. List of primary antibodies used for western blotting of phospho-regulation and protein expression in adipose tissues and liver, see data in figure 2 and 3.
